# Platelet Activating Factor Synthesis and Metabolism in Intestinal Ischaemia-Reperfusion Injury

**DOI:** 10.1155/S0962935194000554

**Published:** 1994

**Authors:** M. J. Mangino, M. Murphy, A. Bohrer, J. Turk

**Affiliations:** 1 Departments of Surgery, Pathology and Medicine Washington University School of Medicine St. Louis Missouri 63110 USA; 2 Division of Critical Care Medicine Miami Children's Hospital Miami Florida 33155 USA

## Abstract

The object of this study was to characterize the synthesis and metabolism of platelet activating factor (PAF) by intestinal mucosa subjected to ischaemia–reperfusion injury. Canine intestinal mucosa produced 16:0-PAF, 18:0-PAF, and high levels of the corresponding lyso- PAF metabolites. Three h of intestinal ischaemia and ischaemia followed by 1 h of reperfusion did not affect the synthesis or metabolism of PAF by intestinal mucosa. Intestinal mucosa elaborated a factor that rapidly hydrolyzes PAF to lyso-PAF. The observed hydrolysis rate was not altered by ischaemia or ischaemia and reperfusion. In conclusion, this study suggests that intestinal mucosa produces PAF and rapidly hydrolyzes PAF. The PAF synthesis and metabolism rates of intestinal mucosa is not altered by ischaemia reperfusion in this model under the imposed conditions.


Mediators of Inflammation 3, 393–395 (1994)

### Platelet activating factor synthesis and metabolism in intestinal ischaemia–reperfusion injury

M. J. Mangino,[CA] M. Murphy, A. Bohrer and J. Turk

Departments of Surgery, Pathology and Medicine, Washington University School of Medicine, St. Louis, Missouri 63110, USA; and Division of Critical Care Medicine Miami Children's Hospital, Miami, Florida 33155, USA

[CA] Corresponding Author

**The object of this study was to characterize the synthesis and metabolism of platelet activating factor (PAF) by intestinal mucosa subjected to ischaemia–reperfusion injury. Canine intestinal mucosa produced 16:0-PAF, 18:0-PAF, and high levels of the corresponding lyso-PAF metabolites. Three h of intestinal ischaemia and ischaemia followed by 1 h of reperfusion did not affect the synthesis or metabolism of PAF by intestinal mucosa. Intestinal mucosa elaborated a factor that rapidly hydrolyzes PAF to lyso-PAF. The observed hydrolysis rate was not altered by ischaemia or ischaemia and reperfusion. In conclusion, this study suggests that intestinal mucosa produces PAF and rapidly hydrolyzes PAF. The PAF synthesis and metabolism rates of intestinal mucosa is not altered by ischaemia reperfusion in this model under the imposed conditions.**

**Key words**; Canine, Ischaemia, Lipid mediators, Mucosal damage

### Introduction

Intestinal ischaemia followed by reperfusion results in characteristic mucosal lesions. Increases in microvascular permeability with transvascular movement of fluid and macromolecules frequently occurs. Although the mechanisms of ischaemic damage are not completely understood, local inflammatory mediators have been implicated in the process. These mediators include oxygen radicals,[1,2] eicosanoids[3] and platelet activating factor.[4,5]

Exogenous PAF increases intestinal capillary permeability and promotes leukocyte adhesion to the post-capillary venous endothelium.[6,7] Ischaemia-induced changes in intestinal permeability are attenuated by *Ginko biloba* extracts which contain PAF receptor antagonist activity,[8] and synthetic PAF receptor antagonists[1] reduce the histological evidence of ischaemic damage. Material extracted from ischaemic intestinal tissue and extracts from post-ischaemic venous blood have been reported to possess PAF-like activity in platelet aggregation assays.[4] Platelet aggregation assays, however, respond to many pro-aggregatory mediators and stimuli, do not provide insight into the chemical PAF species, and do not detect biologically inactive PAF metabolites or inactive PAF precursors. A large amount of indirect and circumstantial evidence implicates PAF in intestinal ischaemia–reperfusion injury, but direct evidence of PAF production in these models is not available, to the best of our knowledge. Therefore, we characterized PAF synthesis and metabolism by intestinal mucosa subjected to ischaemia–reperfusion using physico-chemical techniques, namely gas chromatography–mass spectrometry (GC–MS).

### Materials and Methods

All experiments ($n = 8$) were conducted using anaesthetized dogs (sodium pentobarbitol, 30 mg/kg, i.v.). Segments of the distal ileum were subjected to 3 h of ischaemia by reducing the arterial perfusion pressure to 30 mmHg followed by 1 h of reperfusion under full aortic pressure. Samples of the mucosa were obtained before induction of ischaemia, after 3 h of ischaemia, and after the 1 h reperfusion period. About 300 mg of mucosal tissue was incubated in 2.5 ml Krebs buffer for 30 min and frozen until PAF analysis. Mucosal PAF and lyso-PAF molecular species were quantitated by stable isotope dilution gas chromatography–negative ion chemical ionization–mass spectrometry using deuterium labelled PAF as an internal standard. This technique was developed in this laboratory and is described in detail elsewhere.[9] Blank samples containing no tissue with only the internal standard were included in all analyses. PAF and PAF metabolites were measured as the molecular ion of the pentafluorobenzoate derivative. Lyso-PAF present in the samples was further derivatized to the Sn-2 propionate derivative as described previously.[9]

Additional experiments were conducted to measure the capacity of the incubation media obtained from normal and ischaemic intestinal tissue to hydrolyze PAF to lyso-PAF. Incubation medium (1 ml) was obtained at 30°C and authentic $^{3}$H-

© 1994 Rapid Communications of Oxford Ltd labelled PAF (New England Nuclear, Boston, MA, $^3$H-acetyl label) was added. The reaction mixture was stopped at various times by the addition of 2 volumes of methanol. Lipids, including PAF and lyso-PAF, were extracted from the reaction mixture by the Bligh–Dyer method for $^3$H-content and determined by liquid sciutillation spectroscopy. Hydrolysis of $^3$H-acetyl PAF was determined as the late of loss of $^3$H from the organic phase into the aqueous phase, into which $^3$H-acetate partitions following enzymatic hydrolysis of the added $^3$H-PAF. Nonenzymatic hydrolysis in Krebs buffer not exposed to intestinal tissue was determined and subtracted from all tissue values.

Data are expressed as the mean ± the standard error of the mean and were analysed by analysis of variance with BonFerroni's test for multiple comparison of means. A $p$ value of 0.05 was considered statistically significant.

### Results

The production of 16:0 PAF, 18:0 PAF, 16:0 lyso-PAF, and 18:0 lyso-PAF by intestinal mucosa is shown in Fig. 1. PAF synthesis was determined in normal mucosa, ischaemic mucosa, and mucosa subjected to ischaemia and reperfusion. Although synthesis of PAF and lyso-PAF was clearly measurable, no significant influence of ischaemia or reperfusion, relative to non-ischaemic levels was observed. Interestingly, statistically significantly higher (about 7-fold) amounts of the corresponding 2-lyso-PAF metabolite was detected in all mucosal tissue assayed. The signal attributable to the 18:1-PAF derivative was also monitored, but this material was not detected in any mucosal tissue samples. Clearly, intestinal mucosa produces two 1-O-alkyl species of PAF with the corresponding 2-lyso PAF metabolite being far more abundant (7-fold). Furthermore, synthesis of this mediator was not influenced by tissue ischaemia or reperfusion.

Fig. 2 shows that intestinal mucosa liberates a factor that rapidly hydrolysis exogenous PAF to lyso-PAF. Incubation media that did not contain intestinal mucosa (tissue blank) hydrolyzed PAF to < 5% indicating that the activity was derived from the tissue. This tissue-derived factor released into the incubation media is presumed to be the enzyme PAF acetyl hydrolase. These data also indicate that media derived from ischaemic tissue or from tissue after reperfusion hydrolyze PAF rapidly but not at a different rate than normal mucosa. These data are consistent with the PAF and lyso-PAF levels measured in normal and ischaemic intestinal mucosa (Fig. 1). Lyso-PAF was more predominant than was PAF, and production rates of both PAF and lyso-PAF were independent of ischaemia and reperfusion.

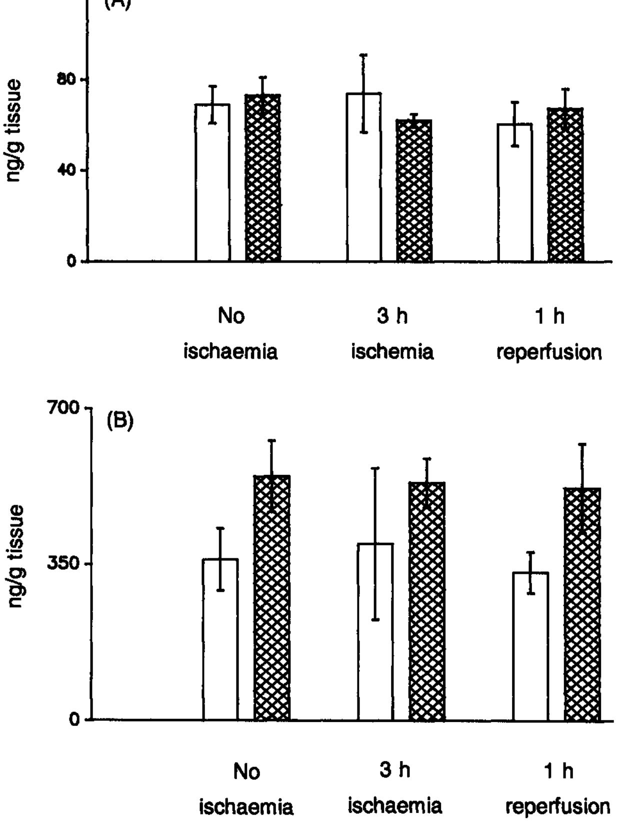

FIG. 1. Synthesis of various molecular species of PAF and lyso-PAF by intestinal mucosal tissue before ischaemia, after 3 h ischaemia, and after 1 h reperfusion. All analytes were measured by stable isotope dilution GC/MS as described in Methods. All results are mean + S.E.M., $n = 8$. Note the dramatic differences in the scales of the upper panel (PAF) and the lower panel (lyso-PAF). Panel (A): □, 16:0 PAF; ⊠, 18:0 PAF. Panel (B): □, 16:0 lyso-PAF; ⊠, 18:0 lyso-PAF.

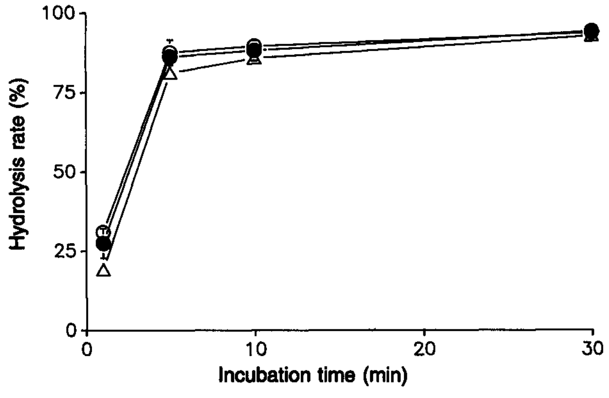

FIG. 2. The rate of PAF hydrolysis to lyso-PAF of incubation media obtained from incubations containing intestinal tissue before ischaemia, after 3 h ischaemia, and after 1 reperfusion. Nonspecific PAF hydrolysis, calculated from the hydrolysis of PAF in media without intestinal tissue, was less than 5% and has been subtracted from the rates shown for intestinal tissue, $n = 8$, Key: (O), before ischaemia; (●), ischaemia; (△), reperfusion.

### Discussion

This study uses sensitive GC/MS techniques to characterize PAF synthesis and metabolism in intestinal mucosal tissue subjected to ischaemia–reperfusion injury. The results clearly indicate that intestinal mucosa synthesize PAF and, to a much greater extent, lyso-PAF. However, the production of PAF was not affected by ischaemia or ischaemia–reperfusion in this particular setting. Also, mucosal tissue very rapidly inactivates PAF to lyso-PAF at equal rates in normal and ischaemic intestine.

The time frame of PAF synthesis and metabolism in intestinal ischaemia–reperfusion injury should be considered. This study examined mucosal synthesis and metabolism of PAF after 1 h of reperfusion. Generally, it is recognized that reperfusion injury following cellular ischaemia occurs shortly after the reperfusion period. A 1 h post-reperfusion sampling period was chosen in this study since significant changes in mucosal arachidonate metabolism after 3 h of intestinal ischaemia, and after 1 h of reperfusion have been demonstrated previously in this laboratory.[3] The possibility that alterations in PAF synthesis or metabolism occur at earlier or later time points from the designed 1-h reperfusion sampling period cannot be ruled out from this study, and should be considered.

Intestinal ischaemia and reperfusion results in cellular inflammation with the infiltration of neutrophils into the tissue.[2] The synthesis of arachidonic acid metabolites also increases after intestinal ischaemia–reperfusion, suggesting a pro-inflammatory role for lipid mediators in this condition.[3] Since arachidonic acid metabolism and PAF synthesis often occur concomitantly, investigations directed at understanding an involvement of PAF in the functional deterioration and cellular inflammation associated with intestinal ischaemia–reperfusion has been undertaken. Platelet activating factor administration produces bowel necrosis and induces a condition similar to splanchnic artery occlusion shock when administered to experimental animals.[10] Platelet activating factor is also involved in ischaemia-induced adherence of leukocytes to post-capillary endothelium in the small intestine.[6] Also, a platelet aggregating material has been extracted from cat intestinal mucosa after ischaemia and reperfusion[6] and a similar aggregating material was obtained from intestinal venous blood in dogs after reperfusion.[4] Taken together, these studies provide circumstantial evidence that PAF is released during intestinal ischaemia and that PAF may play a causal role in the disease. The present data, however, do not indicate any changes in mucosal PAF synthesis or metabolism after intestinal ischaemia or reperfusion (Fig. 1). The physico-chemical GC/MS methods used in this study leave little room for error in interpreting the results, unlike platelet aggregation bioassays. Ischaemia-induced PAF synthesis was not observed in this study, and may represent species differences or the methods used to induce splanchnic ischaemia in other studies. Other studies linking intestinal ischaemia with PAF, induce ischaemia indirectly, often by administering other mediators such as tumour necrosis factor,[5] endotoxin,[11] and even PAF itself.[10] The involvement of PAF in these models of intestinal ischaemia may, therefore, be secondary to other factors inherent in the model itself. These factors may include cytokine or LPS-induced PAF synthesis by resident cells in the gut. Therefore, intestinal ischaemia–reperfusion injury can result from many causes, some may stimulate intestinal PAF synthesis while others do not. This study indicates that the mechanical obstruction of blood flow to the small intestine does not influence the rate of PAF synthesis or metabolism in this model.

### References

1. Droy-Lefaix MT, Drovet Y, Geraud G, Braquet P. Effect of the platelet-activating factor (PAF) antagonist, BN 52021, on free radical-induced intestinal ischemia–reperfusion damage in the rat. In: Emerit, I (ed). *Antioxidants in therapy and preventative medicine.* New York: Plenum Press, 1990; 419–422.
2. Grisham M, Hernandez L, Granger DN. Xanthine oxidase and neutrophil infiltration in intestinal ischaemia. *Am J Physiol* 1986; **251**: G567–G574.
3. Mangino MJ, Anderson CB, Murphy M, Brunt E, Turk J. Mucosal arachidonate metabolism and intestinal ischemia–reperfusion injury. *Am J Physiol* 1989: G299–G307.
4. Filep J, Herman F, Braquet P, Mozes T. Increased levels of platelet-activating factor in blood following intestinal ischemia in the dog. *Biochem Biophys Res Commun* 1989; **158**: 353–359.
5. Sun X, Hsueh W. Bowel necrosis induced by tumor necrosis factor in rats is mediated by platelet-activating factor. *J Clin Invest* 1988; **81**: 1328–1331.
6. Kubes P, Ibbotson G, Granger DN. Role of platelet-activating factor in ischemia/reperfusion-induced leukocyte adherence. *Am J Physiol* 1990; **259**: G300-G305.
7. Kubes P, Suzusi M, Granger DN. Platelet-activating factor-induced microvascular dysfunction: role of adherent leukocytes. *Am J Physiol* 1990; **258**: G158–G163.
8. Otamirt T, Tagesson C. *Ginkgo biloba* extract prevents mucosal damage associated with small-intestinal ischemia. *Scand J Gastroenterol* 1989; **24**: 666–670.
9. Turk J, Bohrer A, Stump T, Ramanadham S, Mangino MJ. Quantification of distinct molecular species of the 2-lyso metabolite of platelet-activating factor by gas chromatography–negative-ion chemical ionization mass spectrometry. *J Chromatography* 1992; **575**; 183–196.
10. Hsueh W, Gonzalez-Crussi F, Arroyave JL. Sequential release of leukotrienes and norepinephrine in rat bowel after platelet-activating factor. *Gastroenterology* 1988; **94**: 1412–1448.
11. Boughton-Smith NK, Hutcheson I, Whittle BRJ. Relationship between PAF acether and thromboxane $A_2$ biosynthesis in endotoxin-induced intestinal damage in the rat. *Prostaglandins* 1989; **38**: 319–333.

ACKNOWLEDGEMENTS. We are grateful to Laraine Reed and Linda Christoph for preparing this manuscript. This work was supported by a new investigator award from the National Institutes of Health to M. Mangino (GM-44252).

**Received 7 April 1994; accepted 30 June 1994**
